# P-143. Oral Vancomycin Prophylaxis to Control an Outbreak of Clostridium difficile Infection in ICU Patients in a Large Urban Safety Net Hospital

**DOI:** 10.1093/ofid/ofaf695.370

**Published:** 2026-01-11

**Authors:** Anjali Kewalramani, Shaurya Sharma, Katrina Sandejas, Daniel Rampersad, Zhong Qu, Leon Boudourakis, John Quale, Briana Episcopia

**Affiliations:** SUNY Downstate Medical Center, Brooklyn, NY; SUNY Downstate University of Health Sciences, Brooklyn, NY; NYC Health + Hospitals/Kings County, Queens, NY; NYCHHC, Brooklyn, New York; KCHC, Garden City, New York; KCHC, Garden City, New York; NYC Health + Hospitals/Kings County, Queens, NY; NYC H+H/ Kings County, Garden City, New York

## Abstract

**Background:**

Hospital-onset Clostridioides difficile infection (HO-CDI) is associated with significant clinical and economic consequences. Use of broad-spectrum antibiotics for patients in the Intensive Care Units (ICUs) is the leading risk for HO-CDI. Hospital outbreaks may still develop despite comprehensive antimicrobial stewardship programs and infection control measures.

**Methods:**

In response to a large tertiary care urban hospital CDI outbreak in the adult medical and surgical ICUs, a program of primary oral vancomycin prophylaxis (OVP) for select high-risk patients was initiated. Primary prophylaxis was administered for patients ≥ 60 years of age, hospitalized for ≥ 7 days, and receiving a beta-lactam, fluoroquinolone, and/or clindamycin. The Outbreak Period was from Oct 1, 2023 through Mar 15, 2024. The Prophylaxis Period started Mar 16, 2024 and terminated on Aug 31, 2024; a Follow-up Period then was from Sep 1, 2024 to Feb 28, 2025. Patients outside the ICUs were not administered OVP. The number of cases of incident HO-CDI and vancomycin-resistant enterococci (VRE) cases were gathered; rates of infection were determined using the number of patient-days for the ICUs and for the remainder of the hospital during each time period.

**Results:**

In the ICUs during the Outbreak Period, there were 1.91 HO-CDI cases/1000 patient-days. During the Prophylaxis Period, this fell to 0.38 cases/1000 patient days (P=.02). This decrease in HO-CDI cases in the ICUs was sustained during the 6-month Follow-up Period (0.66 cases/1000 patient days, P=.06 compared to Outbreak Period). For patients outside the ICUs, rates of HO-CDI remained unchanged during the three periods: 0.20, 0.29, and 0.13 cases/1000 patient days, P=NS). The rate of VRE cases in the ICUs remained stable during the three time periods: 0.70, 1.52, and 0.82 cases/1000 patient days (P=NS).

**Conclusion:**

A restricted protocol of primary OVP helped terminate a sustained outbreak of HO-CDI in ICU patients; the favorable impact continued for 6 months after stopping primary OVP. OVP may be an effective option for preventing and controlling CDI in high-risk settings.

**Disclosures:**

All Authors: No reported disclosures

